# Recent Advances in Bioengineering Bone Revascularization Based on Composite Materials Comprising Hydroxyapatite

**DOI:** 10.3390/ijms241512492

**Published:** 2023-08-06

**Authors:** Yifan Niu, Lei Chen, Tianfu Wu

**Affiliations:** 1 State Key Laboratory of Oral & Maxillofacial Reconstruction and Regeneration, Key Laboratory of Oral Biomedicine Ministry of Education, Hubei Key Laboratory of Stomatology, School & Hospital of Stomatology, Wuhan University, Wuhan 430079, China; 2 Department of Oral Maxillofacial-Head Neck Oncology, School and Hospital of Stomatology, Wuhan University, Wuhan 430079, China

**Keywords:** hydroxyapatite, vascularization, biomaterials, structural characterization, cells and growth factor

## Abstract

The natural healing process of bone is impaired in the presence of tumors, trauma, or inflammation, necessitating external assistance for bone regeneration. The limitations of autologous/allogeneic bone grafting are still being discovered as research progresses. Bone tissue engineering (BTE) is now a crucial component of treating bone injuries and actively works to promote vascularization, a crucial stage in bone repair. A biomaterial with hydroxyapatite (HA), which resembles the mineral makeup of invertebrate bones and teeth, has demonstrated high osteoconductivity, bioactivity, and biocompatibility. However, due to its brittleness and porosity, which restrict its application, scientists have been prompted to explore ways to improve its properties by mixing it with other materials, modifying its structural composition, improving fabrication techniques and growth factor loading, and co-cultivating bone regrowth cells to stimulate vascularization. This review scrutinizes the latest five-year research on HA composite studies aimed at amplifying vascularization in bone regeneration.

## 1. Introduction

**Figure 1:**
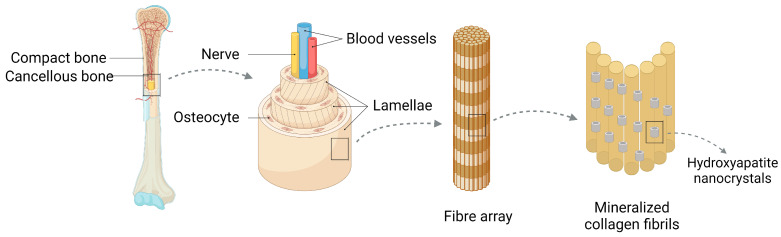
Bone consists of osteoid, periosteum, bone marrow and blood vessels, and lymphatic vessels and nerves, of which osteoid can be divided into osteoid and osteophyte. The osteoid is external, with lamellae approximately three microns thick forming concentric circles around the vertical Haver’s canal containing blood vessels and nerves, forming the functional unit of the bone, while the osteophyte is internal. Hydroxyapatite is involved in the composition of bone as a biomaterial.

Bone is a metabolically active, flexible organ with a special regenerative capacity. The components of bone include bone, periosteum, bone marrow, blood, lymphatic, and nerve cells. Bone can be further subdivided into compact bone and cancellous bone. The osteophysium is located external to the bone, while the vertical Haversian canals that hold blood vessels and nerves are encompassed by concentric circles of lamellae, each approximately three microns wide. The outermost layer of the osteophysium is the periosteum, which consists of fibrous connective tissue. The cancellous bone comprises a network of trabeculae resembling honeycombs, with bone marrow filling the spaces between them [1] (Figure 1). Bone defects are one of the most common injuries, and the number of age-related bone diseases in the United States is expected to increase from 2.1 million in 2005 to 3 million in 2025. In Europe, population growth is predicted to cause an increase in fractures of approximately 28% from 2010 to 2025 [2], and in China, more than 6 million patients suffer from bone defects or dysfunction annually. Clearly, bone loss has a negative impact on patients’ quality of life and represents a significant socioeconomic burden on a global scale [3]. Compared to other tissues, bone faces challenges in repair and regeneration. Conditions such as bone tumor resection, severe trauma, inflammation, or infection can slow down the healing process of the bone, impeding full spontaneous regeneration of the affected area [4]. As a result, external therapies are frequently needed to encourage bone regeneration [5,6].

Bone grafting, categorized as autologous, allograft, and xenograft, remains one of the most commonly used techniques in orthopedic surgery to treat bone tissue deficiencies [7]. Autologous bone grafting is regarded as the gold standard for bone grafting because it combines all the elements necessary for bone regeneration in terms of osteoconduction, osteoinduction, and osseointegration [7,8,9,10]. However, harvesting autologous bone is limited by the amount that can be harvested and results in additional trauma to the patient with associated complications such as pain, bleeding, and infection. [3,4,7,9,11]. Allografts can physiologically restore bone defects without size or shape limitations, preserving the host bone reserve, without damaging the donor site. They also maintain the features of osteoconductivity and osteoinductivity [11,12,13,14]. Despite these advantages, the potential risks of disease transmission, infection, and immune rejection limit the use of allograft bone grafts in clinical practice [7,13,14,15]. Therefore, there is an urgent need to develop more reliable and durable healing techniques to overcome the limitations of traditional bone grafting.

Bone tissue engineering (BTE) has been recognized as an alternative to bone grafting alternative as the study of bone regeneration has progressed [3,16]. BTE is a cutting-edge interdisciplinary discipline that combines bioengineering, cell transplantation, and materials science to provide a biological alternative for fracture repair. By creating an ideal biomimetic environment that stimulates the regeneration and proliferation of normal tissues and cells, BTE not only heals broken bones, but also overcomes the drawbacks of existing therapeutic treatments [3]. Cells, growth factors, and the biological scaffolds that support them are the essential components needed for tissue engineering. Tissue-engineered composites, which are represented by hydroxyapatite (HA), have emerged as one of the best options for treating bone injuries among the various bone substitute materials [17]. Composite materials are made from two or more distinct components that have properties different from those of the individual components and are often designed to combine the best qualities of each component material while overcoming the defects of the single material. The most stable calcium phosphate derivative at human pH is HA, which also has excellent osteoconductivity and induction properties that promote cell adhesion and proliferation [18]. Its chemical and structural characteristics are similar to those of the inorganic components of bone and teeth [19,20,21,22,23]. The advantages of HA over traditional metal (stainless steel, titanium alloy) and ceramic (alumina, silicon nitride) based graft substitutes are significant. HA has good structural integrity, corrosion resistance, osteoinductivity, osteoconductivity, and biocompatibility. However, the inherent limitations of HA, such as its austere hardness, brittleness, and porousness, impede its optimal functionality. Therefore, in order to overcome the performance deficiencies of HA materials and enhance their overall effectiveness, hydroxyapatite composites were established. Consequently, researchers have pioneered HA composites to bolster the overall efficacy of these biomaterials [5,24].

**Figure 2:**
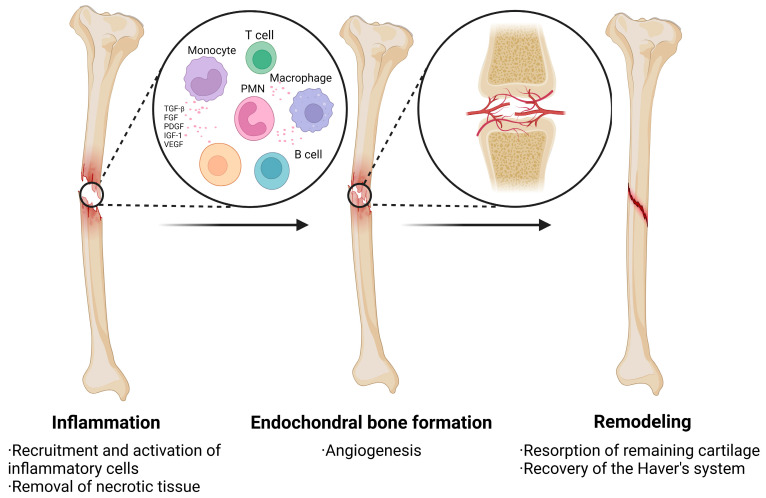
The process of healing after a bone injury is divided into three main phases: inflammation, endochondral bone formation, and remodeling. During the inflammatory phase, bone injury stimulates the release of large amounts of growth factors that promote the migration, recruitment, and proliferation of MSCs. The second phase focuses on the formation of prepared bone with angiogenesis. The final phase is bone remodeling.

Angiogenesis is essential for BTE. Because bone is a highly vascularized tissue that depends on close spatial and temporal connections between blood vessels and osteocytes to maintain integrity, inadequate vascularization frequently causes poor bone regeneration and reduced bone formation when bone replacement materials are implanted in large bone defects [6,25]. Following a bone injury, the body undergoes three primary stages of healing: inflammation, endochondral bone production, and remodeling (Figure 2). During the inflammatory phase, bone damage triggers the release of a significant number of growth factors that promote the migration, recruitment, and proliferation of mesenchymal stem cells (MSCs) as well as the formation of the primary callus. In the second stage, endochondral ossification produces braided bone through the initial differentiation of MSCs into chondrocytes, the cartilaginous precursors of bone [26,27]. Blood vessels develop within the cartilage, causing the cartilage to deteriorate, while invasive MSCs differentiate into osteoblasts, causing the development of new bone [28,29]. Composite materials are made from two or more different components that have properties different from those of the individual components and are often designed to combine the best qualities of each component material while overcoming the defects of the single material.

For MSCs to travel to damaged tissue areas and absorb oxygen and nutrients during this process, sufficient vascularization is a need [3,15]. In addition, sustaining cell viability through perfusion of the healing zone allows for rapid disposal of any cell lineage added to the stroma [15]. It has been demonstrated that during in vitro growth of MSCs, the vasculature, an essential source of replenishment, stimulates the production of bone morphogenetic protein and alkaline phosphatase (ALP) [30,31,32,33,34,35,36]. By releasing paracrine signals, it controls cell proliferation, differentiation, and regeneration, allowing cells of many lineages to communicate and promote tissue healing [37,38,39,40]. The uneven braided bone is modified by osteoclast–osteoblast coupling in the penultimate stage of bone regeneration to become lamellar bone that eventually bridges the fracture [26,27].

In this paper, we review the development of research on vascularization in HA composites for bone regeneration over the past five years, describing the mechanisms and significance of vascularization as well as the effects of materials, structural characterization changes, cell and growth factor interactions, and fabrication processes on HA scaffolds for promoting vascularization.

## 2. Mechanisms by Which HA Composites Promote Vascularization in Bone Regeneration

### 2.1. Mechanisms of Angiogenesis

Blood vessels can proliferate either by sprouting from the terminal ends of pre-existing capillaries or through the mechanism of endothelial progenitor cells (EPCs) present in the healing tissue. The genesis of vascular networks relies heavily on the symbiotic interplay between endothelial cells and the encompassing pericytes [41,42]. Angiogenesis, an elaborate process, requires the orchestrated interaction of diverse cell types and growth factors, both spatially and temporally, to yield robust, stable, and functional blood vessels. This multifaceted process also necessitates the proliferation, differentiation, and migration of endothelial cells, along with the expression of regulatory protein hydrolases.

**Figure 3:**
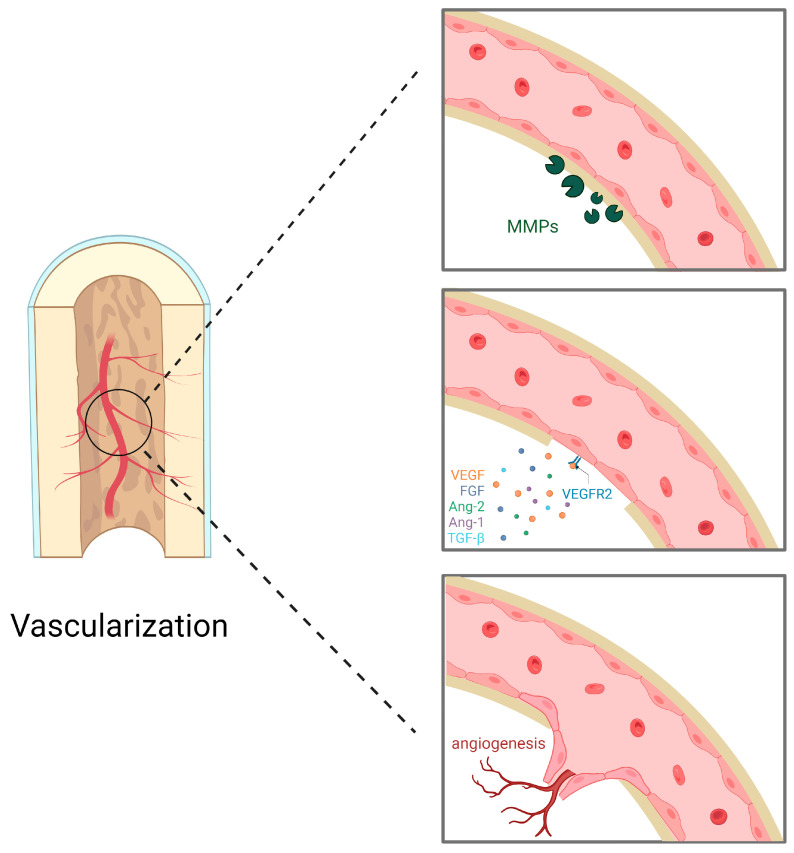
Endothelial cells degrade extracellular matrix metalloproteinases (MMPs). Pro-angiogenic factors, represented by vascular endothelial growth factor (VEGF), bind to receptors on the surface of endothelial cells; these pro-angiogenic factors also include fibroblast growth factor (FGF), angiopoietin-2 (Ang-2), angiopoietin-1 (Ang-1), and transforming growth factor-β (TGF-β). Endothelial cells invade the extracellular matrix, migrate and proliferate, directing the new blood vessels to the stimulus and forming new blood vessels.

Several cells are pertinent to angiogenesis, as well as angiogenesis-facilitating substances are also involved in this process. The initiation of angiogenesis is orchestrated through the collaborative efforts of vascular endothelial growth factor (VEGF), fibroblast growth factor (FGF), and angiopoietin-2 (Ang-2). Endothelial cells, aided by matrix metalloproteinases (MMPs), degrade the extracellular matrix (ECM), reducing pericyte interaction and releasing extracellular matrix-stimulating growth factors. These cells then migrate into the extracellular matrix to proliferate and cause neovascularization [39]. A well-established and mature vascular network emerges through the augmentation of endothelial cell aggregation around new blood vessels, facilitated by platelet-derived growth factors, transforming growth factor (TGF), and angiopoietin-1 (Ang-1) (Figure 3).

Among these, VEGF is a crucial, angiogenesis-specific growth factor and a key promoter of angiogenesis, playing an integral role in its regulation. Thus, the capacity to stimulate VEGF production is considered an important yardstick for evaluating the vascularization potential of a substance. Angiogenesis is modulated by oxygen-disturbing enzymes (at low levels) in the local microenvironment, with enhanced hypoxia-inducible factor (HIF-1), a transcription factor in osteoblasts, prompting downstream transcriptional escalation and consequent upregulation of pro-angiogenic factors. Their activity intricately governs blood vessel infiltration, both temporally and spatially. Hypoxia is posited as the primary inducer of VEGF expression. Endothelial nitric oxide synthase (eNOS) transcription is also stimulated by HIF-1, further promoting angiogenesis and vasodilation [43,44].

VEGFR2 is the primary VEGF signaling receptor, expressed primarily in endothelial cells to mediate angiogenesis and vasculogenesis, as well as to promote vascular permeability in response to VEGF. VEGF binds to VEGFR, which undergoes dimerization, leading to the phosphorylation of certain receptor tyrosine residues and mediating downstream mitogenic, chemotactic, and pro-survival signaling [45].

Angiogenesis represents a cornerstone in the tissue engineering pathway to osteoinduction, where it induces increased osteoclast activity and stimulates osteoblast growth to facilitate new bone formation. Moreover, a synergistic mechanism exists between osteoblast differentiation and angiogenesis. While osteoblasts influence vascular endothelial cells by promoting the synthesis of VEGF and basic fibroblast growth factor (BFGF), thereby further driving angiogenesis, vascular endothelial cells affect osteoblast differentiation through the secretion of an insulin-like growth factor (IGF), prostaglandins, and colony-stimulating factor (CSF). This is a highly dynamic process that responds rapidly to tissue demand [41,46].

### 2.2. The Role of HA Composites in Angiogenesis

In the context of selecting and preparing materials for bone tissue engineering, it is important to take into account the ability of cells to grow, proliferate, migrate, and differentiate [39]. HA scaffolds are notably proficient in facilitating the growth of MSCs, providing them with supportive temporary structures and a three-dimensional milieu conducive to deep cellular proliferation [47]. This allows cells integral to bone injury repair, such as osteoblasts and endothelial cells, to infiltrate the pores and channels of the HA scaffold. The rationale behind this is that the release of calcium and phosphate ions from HA promotes osteogenic development in MSCs [48]. In vivo experiments in nude mice have also shown that hydroxyapatite-containing scaffolds significantly upregulate the expression of osteogenic and angiogenic genes (*Col* I, *ALP*, *OCN*, *VEGF*) and protein secretion (ALP, RUNX2, VEGF) by promoting cell growth [49]. Implantation of the biomaterial into the body triggers an inflammatory response that recruits neutrophils and macrophages from adjacent tissues and circulation to the implantation site. These aggregated inflammatory cells release a lot of chemokines, such as interleukin-1 (IL-1), interleukin-6 (IL-6), interleukin-11 (IL-11), interleukin-18 (IL-18), tumor necrosis factor-alpha (TNF-α), and transforming growth factor beta1 (TGF-β1), enhancing the attraction of MSCs and other cells to the area [50]. Additionally, HA scaffolds assist MSCs in the production and secretion of VEGF, which in turn stimulates angiogenesis via the aforementioned mechanisms [39,48]. As the scaffold degrades, the process of vascularized bone regeneration is completed.

The researchers’ in vivo experiments confirmed the ability of the hydroxyapatite composite scaffold to promote vascularization. A cranial defect model was first established and then the scaffold was implanted in the appropriate location, after which the fluorescence intensity of CD31 was detected, indicating the maturation of the vascular network. A chicken-charged allantoin membrane (CAM) assay was also performed, confirming the good in vivo neovascularization properties of the hydroxyapatite composite scaffold [51].

Additionally, HA complexes gradually release their components during biodegradation, which can stimulate osteogenesis and angiogenesis at the cellular level. In particular, porous HA complexes are more conducive to the adhesion of biological tissue cells and new bone growth due to their larger surface area. During vascularization, these biodegradation products may act as signaling molecules that stimulate the proliferation and migration of vascular endothelial cells, thereby promoting the formation of new blood vessels. However, the rate of biodegradation of HA complexes may be influenced by a number of factors, including their content in the complex, the structure of the complex, and the biodegradation protocol. These factors may affect the rate and amount of product released during the biodegradation of HAp complexes and thus their efficiency and effectiveness in inducing angiogenesis. Overall, the biodegradation of HA complexes may be an important mechanism for their induction of angiogenesis. Modifying the biodegradable nature of HAp complexes may help to optimize their performance in biomedical applications, including applications in bone tissue engineering and angiogenesis. However, more research is needed to further reveal the detailed correlation between biodegradation and angiogenesis of HAp complexes and how the angiogenic effect can be optimized by modulating the biodegradation process.

## 3. Diverse Approaches to Augment the Vascularization-Promoting Attributes of HA Scaffolds

While HA stents offer numerous advantages, they inevitably manifest certain drawbacks as an individual material. To counteract these limitations, such as low stiffness, brittleness, and minimal porosity that contribute to vascularization insufficiencies, researchers have deployed a range of tactics to enhance the functionality of these stents. A prevalent strategy involves the integration of varied substances, encompassing inorganic elements, natural polymers, and synthetic polymers, to enhance the respective strengths of each. Furthermore, modifications in structural characterization, cellular involvement, and growth factors considerably enhance the pro-vascularization properties of HA scaffolds. The potency of these scaffolds can also be magnified through the implementation of refined construction techniques.

### 3.1. Synthesis of Composites through the Fusion of HA with Diverse Materials

**Figure 4:**
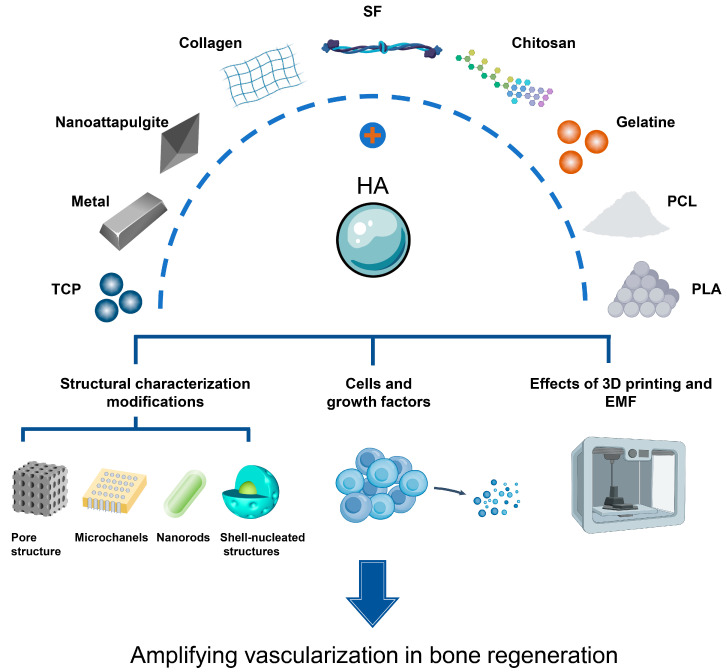
Hydroxyapatite (HA) binds to various organic and inorganic materials to promote the process of bone regeneration vascularization.

Hydroxyapatite composites are hydroxyapatite combined with different polymers and crosslinkers in the form of composites [33]. Numerous composite scaffolds have been revealed to possess potent osteogenic and pro-vascularization traits, and an escalating number of biomaterials have shown considerable potential in the realm of bone tissue engineering in recent years. When integrated with HA, a range of these constituents—including inorganic elements, as well as natural and synthetic polymers—exhibit enhanced properties that promote vascularization (Figure 4).

#### 3.1.1. Inorganic Materials (Table 1)

##### Tricalcium Phosphate (TCP)

Tricalcium phosphate (TCP) is a bioceramic material similar to the naturally occurring HA found in hard tissues, known for its commendable osteoinductive and degradative properties. With phosphate ion subunits and calcium ions balanced in hard tissue engineering, this regulatory framework possesses the capacity for bone repair, positioning TCP as a superior graft material for bone regeneration in tissue engineering. At present, TCP is widely used in the medical field. Despite its abundant beneficial traits, TCP has certain limitations, including its extreme difficulty in unstable transport due to insufficient compressibility and considerable instability [52]. Consequently, the incorporation of polymers becomes an inevitable choice. and HA is often mixed up with TCP. Biphasic calcium phosphate (BCP) ceramics, which are another name for HA/TCP biomaterials, have a variety of unique physical and chemical characteristics, including a high HA/TCP ratio, macroporosity, dual porosity, and an interconnected structure [41]. The pro-angiogenic capability of this biphasic calcium phosphate ceramic is intrinsically tied to its superior osteogenic potential, which exceeds that of both HA and TCP [53].

To decipher the angiogenesis process within BCP ceramic, the researchers used utilized a transwell model, revealing that MSCs navigate purposefully toward BCP. Real-time PCR (RT-PCR) was employed to analyze the gene expression of VEC-related factors, such as KDR (vascular endothelial growth factor receptor-2, VEGFR-2), von Willebrand factor (vWF), vascular cell adhesion molecule-1 (VCAM-1), and calmodulin 5 (CDH5).

The results demonstrated that, in response to stimulation by BCP ceramics, MSCs genetically develop into endothelial cells, with the expression of all four hallmark factors significantly higher than in the control group. Further scrutiny of the intracellular and extracellular protein expression of the KDR, vWF, VCAM-1, and CDH5 vascular endothelial cell division components was conducted using ELISA kits. This confirmed that the expression of all four distinguishing factors was significantly higher than that of controls, supporting the gene expression data that showed MSCs differentiate toward endothelial cells at the protein level in response to stimulation by BCP ceramics [50,54,55]. Collectively, this evidence supports the proposition that BCP promotes angiogenesis.

**Table 1:** Comparison of advantages and disadvantages of inorganic materials.

Inorganic Composition	Advantages	Disadvantages	References
Tricalcium phosphate	Good osteoinduction and degradation	Difficulty in transporting to the correct locationdifficult to compress sufficiently fragile	[52,53,56]
Metal	Good ability to induce angiogenesis	Secondary infection caused by corrosion cytotoxicity due to high concentrations Stress shielding due to excessive metal mechanical strength	[43,57]
Nanoattapulgite	Good HUVEC affinity	/	[58]

##### Metallic Doping

Despite the low trace element concentrations in bone, their absence can directly influence bone formation and metabolism, raising the risk of orthopedic diseases due to diminished bone quality. Consequently, the notion of doping HA materials with bioactive metals to create composites, inspired by the trace elements in natural bone, has garnered significant interest. When metallic materials are employed independently as scaffolds, they risk secondary infections from corrosion, cytotoxicity from high metal ion release, and stress shielding from excessive metallic mechanical strength, all of which can catalyze bone resorption [57]. Contrarily, the incorporation of bioactive metals into HA materials eradicates these detriments and fosters angiogenesis. Research indicates that Cu^2+^, Fe^3+^, Mg^2+^, Sr^2+^, and Yb^2+^ can all effectively stimulate angiogenesis, with the elevation of angiogenesis-related factors serving as the primary mechanism (ion-doping) [43,59]. By using volumetric rate thermal reaction and low-temperature rapid prototyping techniques, the researchers created a number of the aforementioned metal-doped scaffolds. By comparing them to the original scaffolds, they then determined whether Cu^2+^ loading could stimulate endothelial cell differentiation and proliferation, while also upregulating HIF-1α by simulating hypoxia. Fe^3+^ loading enhanced VEGF-A and HIF-1α expression and promote NO secretion and eNOS product.

As a result, the number of vessels considerably rises and new microvessels proliferate more rapidly. Mg^2+^ loading also promotes angiogenesis by increasing VEGF-A expression and also plays a crucial role in controlling the transition of polarized macrophages to an M2 phenotype [52,60,61]. MSCs are attracted to the region of the defect by M2-type macrophages, which also express high levels of VEGF, IL-10, and eNOS, and promote fibroblasts’ extracellular matrix deposition and endothelial cells’ angiogenesis [62,63,64]. By increasing VEGF-A expression, Sr^2+^ and Yb^2+^ loading also escalate angiogenesis and endothelial cell growth [65,66].

##### Nanoattapulgite

Nanoattapulgite, a nanoscale hydrated layered magnesium silicate mineral, is characterized by a rod-like crystalline structure and distinctive physicochemical properties. These include high viscosity, a large specific surface area, an exceptional absorption capacity, and abundant natural reserves [58]. Additionally, it has been suggested that the incorporation of nanoattapulgite into composite scaffolds can yield extremely viscous network architectures through rod–rod interactions, potentially facilitating osteogenesis [67].

Human umbilical vein endothelial cells (HUVECs) were cultured with or without nanoattapulgite in HA extracts for 12 h to examine the angiogenic effects of nanoagglomerate in HA scaffolds. Staining results indicated that HA scaffolds infused with nanoattapulgite had a good affinity for HUVECs and promoted the growth of additional capillaries and vascular networks. Additionally, the scratch and transwell test findings demonstrated that HUVECs migration improved as the fraction of nanoattapulgite in the scaffold rose [68]. Quantification of the number of nodes, grids, tubes’ overall length, and branching also showed that nanoattapulgite-containing scaffolds stimulated angiogenesis more than other types of scaffolds. The researchers also used RT-qPCR to measure the expression of genes associated with angiogenesis (*HIF-1*, *ENOS*, *FGF*, and *VEGF*). The results showed that vascularization was promoted by the production of those genes, which were considerably increased in the nanoattapulgite-containing group compared to the nanoattapulgite-free group [68].

#### 3.1.2. Natural Polymers (Table 2)

##### Collagen

Human bone tissue naturally contains collagen, which is also abundant in the ECM. Type I collagen fibrils constitute the majority of the organic phase of the bone ECM [69]. Levorotatory triple alpha helices, which are the defining structural feature of type I collagen, are surrounded by additional non-helical structural domains known as terminal peptides to produce procollagen molecules. Osteoblasts are the primary producers and secretors of these procollagen molecules, which self-assemble into supramolecular hierarchical structures to generate protofibrils [69].

Collagen can be cleaved by metalloproteases in the body and has high biocompatibility, osteoconductivity, low antigenicity, and good hydrophilic characteristics [70,71]. It is easy to process and can be added to other biomaterials to improve their properties. The incorporation of collagen also enhances osteoblast function and promotes better biointegration of cells with surrounding tissues. It boosts alkaline phosphatase (ALP) activity and matrix mineralization [70]. Bone marrow mesenchymal stem cells (BMSCs) and HUVECs can attach to surfaces, group together, and carry out their various bioactive functions, according to in vitro research. Live/dead cell staining results further substantiate the superior biocompatibility of the collagen-HA scaffold, favoring cell development and fostering osteogenesis and vascularization [72,73]. Therefore, scaffolds formed by hydroxyapatite and collagen together have promising applications in artificial bone graft substitutes and tissue engineering scaffolds [74].

##### Silk Fibroin (SF)

The biopolymer known as silk fibroin (SF), which is sourced from domestic silkworms, is common and non-irritating. It has a long history of use as a suture material in the human body. Due to its low inflammatory response, high biocompatibility, elasticity, mechanical strength, and regulated biodegradability [75]. With a deeper understanding of these inherent qualities, SF has been fabricated into scaffolds, hydrogels, or nonwovens, and electrostatically spun scaffolds of filamentous proteins have been proved in numerous studies as reliable synthetic bone substitutes for biomedical engineering. However, SF materials on their own do not exhibit osteoinductive activity. This limitation significantly limits the use of SF in bone repair. Additionally, residual filamentous proteins present in the material can contaminate it and cause biocompatibility issues. However, these problems are mitigated when SF forms composites with hydroxyapatite [75,76]. Both the arrangement of the SF molecular chain and HA’s nucleation capability can contribute to the orderly alignment of the SF molecular chain. The interaction between the two enhances the osteogenic activity of the composite scaffold and effectively compensates for HA’s deficiencies. Immunohistochemical studies have shown that angiogenesis is also prevalent in areas with active bone remodeling due to the correlation between angiogenesis and bone regeneration processes. Thus, the combination of both elements can be viewed as a compelling strategy for tissue-engineered bone regeneration scaffolds [77,78].

##### Chitosan and Gelatine

Chitosan (CS) is a desirable biomaterial because it is derived from chitin, the second most abundant and renewable polysaccharide in use today. Chitosan is a naturally occurring cationic polymer that is non-irritating, non-toxic, biocompatible, and degradable. It is a rare positively charged alkaline polysaccharide that has high antibacterial capabilities and the ability to bind to DNA, making it useful for use in the medical industry [19]. Gelatin, a macromolecular hydrocolloid with high surface activity and viscosity, typically does not require the addition of surfactants to achieve low surface tension in its concentrated solutions [79]. In addition, gelatin has natural holes that can hold cells [80]. However, both have poor mechanical and osteoconductive properties, making it challenging to use them alone as a component of a scaffold for bone tissue synthesis. Complementing HA with other materials can improve the performance of HA. Researchers have developed chitosan, gelatin, and HA microgels by emulsifying water and oil. Electron micrographs reveal that these microgels allow cells adhere firmly to the surface of the microgrids and promote the germination of endothelial cells to form a vascular network, which in turn promotes bone regeneration [81].

**Table 2:** Comparison of advantages and disadvantages of natural polymers.

Natural Polymers	Advantages	Disadvantages	References
Collagen	Good hydrophilicity Good biocompatibility	/	[69,70,71]
Silk protein	Good biocompatibility Tough mechanical properties Biodegradable and non-toxic Water vapor permeability Very low inflammatory response	Residues may cause contamination	[75,76]
Chitosan	Good biodegradability Good biocompatibility Non-toxic Non-irritating Antibacterial	Poor mechanical properties Poor osteoconductivity	[19,81]
Gelatine	High surface activity Good viscosity Natural pore space to accommodate cells	Poor mechanical properties Poor osteoconductivity	[79,80,81]

#### 3.1.3. Synthetic Polymers (Table 3)

##### Polycaprolactone (PCL)

As a biocompatible and biodegradable substance with high mechanical strength, polycaprolactone (PCL) has been extensively used in numerous tissue engineering techniques and medical procedures [82]. Researchers have developed PCL microspheres and PCL/HA composite microspheres using a modified solid/oil/water emulsification solvent evaporation method. They then underwent imaging tests, mechanical tests, and histological tests. The results demonstrated that PCL/HA composite microspheres had superior cell adhesion and osteogenic differentiation compared to PCL microspheres. According to in vitro research, PCL/HA composite microspheres encouraged BMSCs to express bone morphogenetic protein 2 (BMP-2) and VEGF, which in turn promoted angiogenesis [83].

##### Polylactic Acid (PLA)

A synthetic polyester with good mechanical properties and biocompatibility, polylactic acid has good degradability and frequently results in sterile inflammatory reactions in the surrounding tissue [84]. To fully stimulate vascularized bone regeneration in light of these constraints, PLA and HA are often combined to form composite biomaterials.

**Table 3:** Comparison of advantages and disadvantages of synthetic polymers.

Synthetic Polymers	Advantages	Disadvantages	References
Polycaprolactone	High mechanical strength Good biocompatibility Biodegradable	/	[82]
Polylactic acid	Good mechanical properties Good biocompatibility	Rapid degradation rates predispose to inflammation	[84]

### 3.2. Structural Characterisation Modifications

#### 3.2.1. Pore Structure

##### Pore Size

Scaffolds with large pores consistently show better osteogenic activity and angiogenesis [85]. In vivo experiments have also shown that angiogenesis in the stent is closely related to the pore size [86]. The pore size of the stent should be at least 100 μm to facilitate cell penetration, internal tissue growth, blood vessel formation, as well as proper regeneration of mineralized bone. In contrast, the pore size should ideally be greater than 200 μm or even greater than 250 μm to facilitate internal bone growth and the colonization and proliferation of osteoblasts within the large pores [87]. Leet et al. found that a scaffold with 500 μm macroporous pores produced more osseointegration and bone regeneration than a scaffold with 250 μm macroporous pores in vivo [88,89,90]. Graphene oxide–hydroxyapatite (GO-HAP) is a 2D nanocomposite prepared by in situ bonding. This compound can form hydrogen bonds with gelatin methacrylate (GelMA) and polyethylene glycol diacrylate (PEGDA) due to the presence of oxygen atoms in GO-HAP. Additionally, the ice crystals act as a pore-forming agent at sub-zero temperatures (−20 °C) by displacing the liquid monomer phase before or during polymerization. This allows GelMA and PEGDA to create macroporous structures that are similar to 3D sponges on the surface of the sulfonated LCFRPEEK which is a brand-new high-performance composite substance. This technique was utilized by the researchers to synthesize cryogels with interconnected macroporous structures that resemble 3D sponges. The durability and the interconnected structure of these constructs present an alternative approach for fostering inward cellular proliferation and migration by crafting vascular networks. HUVECs have the capacity to multiply and navigate within the scaffold, attributable to the substantial volume of water it imbibes and the extent of oxygen and nutrient translocation it allows, culminating in a 3D microvascular construct. Transwell analysis was used by the researchers to create in vitro migration assays to evaluate the impact of different substrates on BMSCs migration, and the results demonstrated that BMSCs were able to adhere, migrate and mineralize well. After 14 days of incubation on diverse samples, the expression of ALP, COL-I, BMP-2, and RUNX2 was increased, indicating that the 3D spongy macropore structure promotes osteogenesis. In the process of bone remodeling, the osteogenesis of stem cells and the angiogenesis of endothelial cells are constantly reinforcing each other. Additionally, GelMA/PEGDA/GO-HAP cryogels on the surface of sulfonated LCFRPEEK implants have improved cytocompatibility in terms of proliferation and diffusion, and the material consequently has prospective applications, according to SEM and CLSM pictures of BMSC and cell proliferation data [91].

##### Pore Number

In general, porous features are necessary for nutrient flow, blood vessel development, and inward cell growth in scaffolds. Porous scaffolds, specifically in tissue engineering, have a significant impact on cell behavior and affect cell adhesion, colonization, and filtration [87]. Porous cell structures significantly increased angiogenic and osteogenic activity as compared to traditional bioprinted collagen/HA cell constructs, according to immunochemistry and gene expression measurements made using reverse transcription-polymerase chain reaction analysis [92,93]. In the in vivo experiments, the scaffolds were implanted into the rat spine after being created using conventional temperature-controlled 3D bioprinting techniques and collagen foaming. The result showed that high porosity cell-loaded structures, compared to traditional bioprinted cell-loaded scaffolds with identical cell types and density, encouraged a noticeable increase in angiogenesis. The research also underscored that a microenvironment with well-connected porous structures could stimulate osteogenesis and vascular differentiation of human adipose stem cells (hASCs) and endothelial cells (ECs). This was driven by the facilitation of effective interactions and communication between these different cell types. This conclusion was substantiated by immunofluorescence staining for osteogenesis and angiogenesis, using osteopontin (OPN) and platelet endothelial cell adhesion molecule-1 (PECAM-1/CD31) antibodies, in standard grid-printed and foam structures, respectively [88,92].

The researchers discovered that all macroporous scaffolds featured bone-like nodules within the scaffold struts and included blood vessels within each macroporous pore. At the synchrotron micro-CT size, there was little interaction between normal bone and HA or demineralized bone matrix (DBM) particles, and the researchers also found that new bone developed exclusively from pre-existing bone. This would also imply that large pore size does not significantly affect the performance of HA/DBM scaffold work for spinal fusion and bone regeneration. Instead, the osteoconductive and osteoinductive properties of the scaffold composite are likely the most important elements in promoting bone formation [88].

##### Pore Distribution

Graded porous scaffolds outperform uniform pore-size scaffolds in bone regeneration, according to previous studies. The researchers created two different types of graded porous scaffolds using the sugar template leaching method: One has large pores of 1100–1250 μm in the center and small pores of 500–650 μm in the periphery (HALS), while the other has small pores in the center and large pores of 1100–1250 μm in the periphery (HASL). According to the in vivo findings, there were differences in the localization of new bone within the defect as a result of the different pore size distributions having a significant impact on angiogenesis during bone development. Although the host vasculature successfully filtered the entire scaffold, one month after implantation, the diameter of the invading vessels surrounding the HASL was significantly larger than the diameter of the center. Three months after implantation, researchers found that new bone was present only at the periphery in HALS, but HASL caused more homogeneous bone growth throughout the bone graft. According to this study, the arrangement of pore diameters in graded scaffolds influences both late bone formation and early angiogenesis. In the healing of significant bone defects, graded scaffolds with large peripheral pores may be able to encourage angiogenesis and osteogenesis. Large pores on the periphery of the HASL provide enough space for host vessels to expand, and a bigger interconnection window makes it easier for them to penetrate. However, only small vessels can be filtered due to the pore size and interconnectivity window diameter in the center of the HASL. For HALS, the maximum diameter of the host vessels that can infiltrate is limited by the small peripheral pores and small interconnecting window dimensions. The smaller diameter capillaries in the filtered peripheral vessels cannot enter the center because of the same blood pressure. Even with the enormous pores, this causes some angiogenesis in the center of the HALS, but little to no new bone growth elsewhere. As a result, the characteristics of the porous scaffold, such as how the pores are distributed throughout the area, the size of the pores, and the size of the interconnecting windows, all work together to determine how many and how deep blood vessels infiltrate it, causing variations in osteogenesis and the distribution of new bone formation in the area [94]. Moreover, the hydroxyapatite composite scaffold with a pore size gradient has strong water absorption, a suitable degradation rate, and good structural stability and is suitable for cell growth and reproduction [95].

#### 3.2.2. Pore Geometry

##### Microchannels

It is commonly acknowledged that porous scaffolds with high porosity and a three-dimensional (3D) porous structure promote improved tissue vascularization and cell penetration. Since it mirrors the pore tissue of the bone unit structure that hosts the vascular network in long bones, the management of pore structure has therefore become a crucial topic in tissue engineering and regenerative medicine over the past few decades [96]. Furthermore, 3D micropatterned pores with controlled pore size and channel-to-channel spacing, colloquially referred to as microchannels, have recently garnered significant interest due to their propensity to promote vascularization within scaffolds. A microchannel is a channel that has extremely small dimensions and a high aspect ratio (typically ranging from a few microns to several hundred microns) [97]. The researchers used a mix of electrostatic spinning, in situ biosynthesis, and laser-assisted perforation techniques to construct microchannels with various pore sizes in 3D cellulose scaffolds. The scaffolds were tested for cell migration using a laser scanning confocal micro-scope (LSCM) five days after inoculation with BMSCs. Cells spread only on the surface of the non-microchannel scaffolds and exhibited minimal migration. All LP-BNC-SCA scaffolds included BMSCs, and the cells were able to migrate via the microchannels. Additionally, the researchers discovered that cells were invisible in the LP-BNC-SCA-3 scaffold with 400 μm microchannels. This suggests that cells cannot be supported by scaffolds with excessively large microchannels, probably because they slide into the bottom of the microchannels. Additionally, LP-BNC-SCA-1 scaffold with 100 μm microchannels had less active cell behavior than LP-BNC-CCA-2, which could be attributed to the tiny microchannels of LP-BNC-SiCA-1. The strongest cell migration was found in LP-BNC-SCA-2 scaffolds with 200 μm microchannels, indicating that microchannel size has a substantial impact on cell survival and migration [98]. Higher cell migration is a requirement for angiogenesis.

##### Shell-Nucleated Structures

To create bone-like structures, researchers developed a bioprinting method based on coaxial micro-extrusion technology. Using a dual-nozzle coaxial microfluidic system coupled with calcium aluminate gel formation and hydrogen peroxide (H_2_O_2_) foaming processes, bioceramic particles with a core–shell structure were finally effectively created [99], which was achieved by simultaneously extruding two different bioinks from the core and shell regions of the coaxial nozzle. The cell-loaded fibers in the bioprinted scaffold have a core–shell or homogeneous structure, allowing for either non-contact (indirect) or contact (direct) with the co-culture of MC3T3 cells and HUVEC. To evaluate the osteogenic and angiogenic activity of HUVECs and MC3T3 cells in bioprinted structures by detecting the expression of endothelial cell-specific markers (*CD31* and *vWF*) and osteogenesis-related marker *OCN*. The results of the angiogenic and osteogenic gene expression analyses showed that both core–shell and homogeneous structures supported the 3D culture of HUVECs and osteoblasts and the expression of osteogenic and angiogenic factors. Gene expression was significantly higher in the core–shell structure than in the homogeneous structure, indicating that the shell–nucleus structure was more conducive to angiogenesis, but this was due to the clear distribution of osteoblasts and endothelial cells and the formation of vascular-like structures in the cell culture system [100].

### 3.3. Cells and Growth Factors

In addition to the above factors, the composition, additional biological factors/cells, and the manufacturing methods are closely related. The vascularization capabilities of hydroxyapatite scaffolds can be directly influenced by cell/growth factor loading, and all of these parameters support vascularization by promoting the migration of pertinent cells or the expression of growth factors.

#### 3.3.1. Growth Factors

##### Vascular Endothelial Growth Factor (VEGF)

The growth and regeneration of bone depend on VEGF, one of the most significant regulators of angiogenesis [101]. VEGF, a component of the natural extracellular matrix in vivo, interacts with sulfated glycosaminoglycans such as heparin or acetyl heparin sulfate. These not only stimulate angiogenesis and attract cartilage debris tissue to hypertrophic cartilage, but also necessitate intramembrane acidification. Notably, extensive VEGF infusions into the defect without a scaffold failed to augment angiogenesis, whereas VEGF loaded into a hydrogel at a remarkably nominal rate enhanced angiogenesis. [102]. In order to sustain its pro-angiogenic effect, VEGF can be loaded into the material and continuously released [103,104,105,106,107,108]. VEGF and vascular endothelial growth factor receptors are heightened at the apex of invasive angiogenic sprouts, and the inhibition of VEGF curtails the expansion of microvasculature [109,110]. In addition to increasing vascular permeability and window opening, VEGF also promotes the release of vWF, integrin and interstitial collagenase expression, and fibrinogen activator and fibrinogen activator receptor expression [110].

The researchers created VEGF-free and VEGF-loaded HA scaffolds, and when they examined the vascularized bone repair by histological staining, they discovered that new red blood cells (RBCs) were detected within the defect area in all groups. Hematoxylin and eosin (H&E) images also revealed the presence of fibrous tissue within the scaffold pores in all groups, which was recognized as a collagen matrix by Masson staining. New bone virtually completely filled up the defects in the VEGF-loaded group [111]. Results from the CCK-8 assay and the transwell assay revealed that VEGF increased endothelial cell growth and migration, resulting in capillary formation [102]. By measuring the levels of OPN, RUNX2, COL-I, VEGFR-2, vWF, and CD31, as well as the expression of Wnt1, LRP-6, and β-catenin, it was found that these proteins and genes were more abundant in the VEGF-free group. Compared to the VEGF-free group, the expression of angiogenesis-related proteins and genes was dramatically increased [112]. It is also vital to remember that too much VEGF might increase vascular permeability, which can cause edema and systemic hypotension. As a result, successful healing of bone injuries requires controlled and sustained release of VEGF [113,114]. In conclusion, HA composite scaffolds supplied with biologically active, sustained-release VEGF promote angiogenesis and aid in bone healing.

##### Bone Morphogenetic Protein-2 (BMP-2)

In addition to its well-documented effects on bone formation, the potent bone morphogenetic agent BMP-2 has also recently been found to increase neovascularization [115,116,117], although the mechanism is under debate. Therefore, scientists have investigated how BMP-2 is delivered to the bone and investigated whether neovascularization is induced directly or indirectly by BMP-2. Several studies have shown that BMP-2 significantly increases only DNA synthesis, but not proliferation, in human aortic endothelial cells (HAEC) and HUVEC. Thus, BMP-2 is not a mitogenic activator of these endothelial cell types, but may serve as a chemoattractant to increase angiogenic cytokines. SEM further revealed that BMP-2 did not stimulate endothelial cell migration or angiogenesis in vitro, but that critical endothelial cell activities for angiogenesis were significantly improved by conditioned media from hMSCs or mouse calvarial osteoblast (COb)-derived cells. Using micro-CT angiography to count and characterize vascular structures within the defects, the researchers demonstrated that BMP-2 treatment dramatically enhanced neointimal volume percentage, vessel number, and vascular connectivity. This indicates that BMP-2-induced angiogenesis does not directly promote endothelial cell activation, but rather occurs through a paracrine process following activation of osteoprogenitor cells. Therefore, BMP-2 delivery alone is largely insufficient for injuries of impaired endogenous cellular origin, such as giant defects or multi-tissue polytrauma. However, in a regenerative setting with an adequate supply of mesenchymal progenitor cells and nearby vessels, BMP-2 may contribute to the induction of bone formation and neovascularization [115].

The U.S. Food and Drug Administration (FDA) has currently only approved BMP-2 for use as a replacement for bone grafts. However, some negative effects have surfaced as the therapeutic use of BMP-2 has grown. In vitro studies have shown that BMP-2 increases the levels of several cytokines and chemokines that cause postoperative inflammation, that BMP-2 leakage outside the implant site may result in the formation of ectopic bone, that osteoclast-mediated bone resorption and inappropriate adipogenesis are linked to signaling pathways related to BMP-2, and that BMP-2 even increases the risk of cancer [118,119].

##### Erythropoietin (EPO)

Stimulation of MSCs to proliferate and differentiate into osteoblasts is thought to be the cause of EPO-induced osteogenesis [110,111,112,113,114,115,116,117,120,121,122]. Additionally, it has been shown to enhance hematopoietic stem cell proliferation and increase BMP-2 synthesis by activating the Janus kinase/signal transducer and activator of transcription (JAK/STAT) signaling pathway to stimulate osteogenic differentiation [123,124]. Other studies have shown that EPO not only increases VEGF expression [125,126], but also physically and functionally associates with VEGF to support blood vessel development and bone repair [127,128,129].

#### 3.3.2. Co-Culture

Endothelial cells (ECs) and supporting cells can be co-cultured to generate prevascular material that can fuse with the host vasculature and enhance perfusion after implantation. With the ability to self-renew and immunosuppress, MSCs with multipotential and homing properties are excellent candidates for cell therapy and enhance angiogenesis by increasing the expression of angiogenesis-related factors [28,80,130,131]. Within one week of implantation, the PSC-EC network survives, which was generated from endothelial cells (ECs) derived from human induced pluripotent stem cells (iPSCs), anastomoses to the host vasculature, and continues to function for eight weeks after implantation. In contrast to scaffolds with only MSC networks, the prevascularized scaffolds stimulated angiogenesis [132]. When bone marrow MSCs and endothelial progenitor cells were co-cultured, the researchers noticed early neovascularization. The indirect transwell co-culture system between EPCs and MSCs allowed the co-cultured MSCs to preserve their stemness without undergoing any morphological alterations. In addition to increased proliferation, co-cultured MSCs showed increased expression of the stemness coregulators *OCT4*, *SOX2*, *Nanog*, and *KLF4*. MSCs were nourished by EPCs to undergo neovascularization and blood perfusion, which delayed the onset of apoptosis. EPCs can survive and develop a reliable vascular network because smooth muscle progenitor cells (SMPCs) secrete angiopoietin-1 (Ang-1), which then activates the receptor Tie-2 on EPCs. Hudo et al. also created an SMC-EPC bilayer cell sheet with spatial orientation and temporal arrangement in UpCell dishes, preserving cell junctions and extracellular matrix. Cell sheet engineering using UpCell dishes is a modern technique that allows for the rapid formation of cell monolayers without the use of ECM enzymatic digestion. It is hydrophilic when the temperature is reduced to allow cell separation without altering cell-cell junctions and ECM deposition [133]. SDF-1, VEGF, HGF, and TGF- were all produced by these cell sheets [134] and increased the activation of FLK1 and VEGFR-2. When combined, SMCs and endothelial progenitor cells produce favorable interactions that promote the growth of functional neovascularization [135].

This was confirmed by in vivo experiments in which hydroxyapatite scaffolds containing stem cells were transplanted subcutaneously into immunocompromised mice. After a 12-week transplantation period, histological analysis showed that vascularized tissue formed in all stem cell-containing constructs [136].

HUVECs are one of the most frequent sources of endothelial cells, and they are crucial for angiogenesis [43]. The current study provides evidence that co-culturing Wharton’s jelly mesenchymal stem cells (WJ MSCs) or hASCs with HUVECs promotes angiogenesis, as evidenced by a significant increase in the expression of *CD31* and *VEGF*, indicators of angiogenesis [137,138,139].

### 3.4. Effects of 3D Printing and Electromagnetic Fields (EMF)

Electromagnetic fields (EMFs) were first utilized to stimulate osteogenesis in 1974 by Bassett et al. Subsequently, there has been a significant amount of study on the use of EMFs to treat bone regeneration [111]. In recent years, scientists have discovered a new breakthrough in the field of increasing bone regeneration vascularization: the combination of safe, non-invasive magnetic therapy and 3D printing. The combination of EMFs and 3D printing promoted the growth and differentiation of BMSCs grown on HA composite scaffolds, in part by activating the ERK and JNK pathways, which are connected to MAPK [111,140].

The researcher used a growth medium to collect bone marrow mesenchymal stem cells (BMSC) by flushing bone marrow from rat femur and tibia in order to study the osteogenic differentiation capacity of EMF on BMSC cultured on scaffolds. Cells were passaged in 5% CO_2_ at 37 °C. The BMSC were then divided into two groups: the osteoinductive medium (OIM) group, in which the cells were cultured in OIM alone, and the OIM+EMF group, in which the cells were both cultured in OIM and subjected to a 1 mT, 15 Hz EMF for four hours each day. RUNX2 and OPN levels increased following EMF therapy according to Western blotting, which was used to identify proteins involved in bone formation. Inoculated cells were cultured in serum-free OIM for 8 h before being treated with or without EMF (1 mT, 15 HZ) for 30 min to examine the potential mechanism of EMF on the bone-forming differentiation potential of scaffold-cultured BMSCs. Later, protein blotting was used to determine whether the MAPK pathway was activated. The results showed that EMF treatment significantly increased the phosphorylation of ERK and JNK. Additionally, angiogenesis at the site of the bone defect was evaluated by micro-CT imaging after the MICROFIL substance was injected into the capillaries. At 6 weeks, there were more angiogenesis and communication branches in the stent/BMSC/EMF group. Then, using VGStudio software, an additional quantitative examination of the newly created vessels was carried out. The control group showed very marginal vessel formation, while the stent/BMSC/EMF group had the greatest vessel area and number. A final histologic evaluation of bone development in the defect in the skull was carried out. By using HE staining to measure new bone development in the different groups’ bone defects at 4 and 12 weeks after implantation, it was found that the scaffold/BMSCs/EMF group had improved bone regeneration and the highest level of biomechanical properties. In conclusion, EMF has a lot of potential for usage in bone regeneration and vascularization when paired with 3D-printed scaffolds [140].

## 4. Summary and Future Prospects

Methods to promote bone regeneration, particularly vascularization, are becoming increasingly sophisticated and well-established, from bone grafting to bone tissue engineering. According to a review of the literature, HA, which is commonly used in bone tissue engineering, is highly similar to natural bone. However, due to its hardness, brittleness, and porosity, pure HA is not as successful as it may be in promoting vascularization, so researchers have used a variety of techniques to enhance the performance of HA scaffolds to promote vascularization in bone regeneration.

Researchers have discovered that HA composites made of inorganic materials (tricalcium phosphate, Cu^2+^, Fe^3+^, Mg^2+^, Sr^2+^, Yb^2+^, and other metal dopants, concave and convex nanorods), natural polymers (collagen, sericin, chitosan, and gelatin) and synthetic polymers (polycaprolactone and polylactic acid) have shown good efficacy in promoting vascularization. We have also learned from these investigations that more and more materials with desirable qualities are being created, opening up new opportunities for bone tissue engineering to repair bone abnormalities. The performance of HA scaffolds has also been significantly impacted by changes in their structural characterization, with macroporous, porous, and hierarchically dispersed scaffolds, as well as microchannel and shell nuclei structures, all promoting angiogenesis. The pore structure has long been a significant problem in bone tissue engineering and is crucial for angiogenesis. In recent years, novel structures with pro-vascularization effects have been found. Second, since cells and growth factors play an important role in angiogenesis, researchers have discovered that HA scaffolds that have been loaded with growth factors or co-cultured with them significantly improve their capacity to promote angiogenesis. This is especially true for vascular endothelial growth factor, which plays an important role as one of the most crucial regulators of angiogenesis and directly influences angiogenesis. As the field evolves, other elements have been identified as active contributors to angiogenesis, including erythropoietin and bone morphogenetic protein-2. Additionally, the production of scaffolds using electromagnetic fields in combination with 3D printing has great potential for use in bone regeneration vascularization.

All of the aforementioned techniques have successfully enhanced the inadequate vascularization and performance of HA scaffolds, encouraged intricate interaction between osteoblasts and angiogenic cells, and offered a dependable support system for the advancement of tissue engineering scaffolds. However, because the majority of these studies used cellular or animal models, they were limited in their ability to be applied in clinical settings. A combination of pharmacological cues, flexible spatiotemporal horizons, appropriate cellular responses, and precise control of overgrowth factor indulgences will also be essential in future investigations to demonstrate the validity of these techniques. It is anticipated that more HA-related biocomposites with greater performance and cost-effectiveness will be developed as the technology develops and changes, with developing technologies such as new nanomedicine precision medicine.

## Data Availability

Not applicable.
